# Semiconductor nanowire metamaterial for broadband near-unity absorption

**DOI:** 10.1038/s41598-022-13537-y

**Published:** 2022-06-11

**Authors:** Burak Tekcan, Brad van Kasteren, Sasan V. Grayli, Daozhi Shen, Man Chun Tam, Dayan Ban, Zbigniew Wasilewski, Adam W. Tsen, Michael E. Reimer

**Affiliations:** 1 Institute for Quantum Computing, University of Waterloo, Waterloo, ON Canada; 2 Department of Electrical and Computer Engineering, University of Waterloo, Waterloo, ON Canada; 3 Waterloo Institute for Nanotechnology, University of Waterloo, Waterloo, ON Canada; 4 Department of Chemistry, University of Waterloo, Waterloo, ON Canada; 5 Centre for Advanced Materials Joining, University of Waterloo, Waterloo, ON Canada; 6 Department of Physics and Astronomy, University of Waterloo, Waterloo, ON Canada

**Keywords:** Metamaterials, Nanowires, Materials for optics, Metamaterials, Metamaterials, Nanowires, Single photons and quantum effects, Sub-wavelength optics, Metamaterials, Nanowires, Photonic devices, Optical physics, Sub-wavelength optics, Engineering, Materials science, Nanoscience and technology, Optics and photonics

## Abstract

The realization of a semiconductor near-unity absorber in the infrared will provide new capabilities to transform applications in sensing, health, imaging, and quantum information science, especially where portability is required. Typically, commercially available portable single-photon detectors in the infrared are made from bulk semiconductors and have efficiencies well below unity. Here, we design a novel semiconductor nanowire metamaterial, and show that by carefully arranging an InGaAs nanowire array and by controlling their shape, we demonstrate near-unity absorption efficiency at room temperature. We experimentally show an average measured efficiency of 93% (simulated average efficiency of 97%) over an unprecedented wavelength range from 900 to 1500 nm. We further show that the near-unity absorption results from the collective response of the nanowire metamaterial, originating from both coupling into leaky resonant waveguide and transverse modes. These coupling mechanisms cause light to be absorbed directly from the top and indirectly as light scatters from one nanowire to neighbouring ones. This work leads to the possible development of a new generation of quantum detectors with unprecedented broadband near-unity absorption in the infrared, while operating near room temperature for a wider range of applications.

## Introduction

The field of optical metamaterials demonstrates promise because of the inherent advantages they exhibit over their bulk counterpart for enhanced absorption or realizing an exotic optical response, which originates from the specific arrangement and the collective interactions of the so-called metaatoms^1–7^. A metaatom is considered the unit cell of a metamaterial, comprised of a single or many structures, whereby their unique arrangement leads to an optical response different from the individual structure. Their coordinated design and careful placement can lead to a near-unity absorber for a broader range of applications. It has been shown that the properties of propagating photons, such as polarization and refraction, through interaction with metamaterials can be exploited to create super-lenses, negative refraction, asymmetric transmission, and cloaking^1–10^. Early demonstrations of such metamaterial properties were achieved by exploiting plasmons (i.e., the collective oscillation of electrons in metals) to control the interaction of light with the surface^1,2,10–12^. In this class of plasmonic metamaterials, the electric dipole response is controlled by the shape, size, and orientation of the metaatoms in the crystalline structure^10–14^. More recently, dielectric and semiconductor metamaterials were used to demonstrate light manipulation with lower losses than their plasmonic counterpart^1,2,15–18^. Indeed, the freedom to control the magnetic and electric field response in such structures leads to the realization of highly absorbing or transmissive metamaterials with unique qualities that are highly desired in sensing and imaging applications^3,5,8,9,15,19–23^.

Near-unity absorption in semiconductor metamaterials has been previously demonstrated by overlapping both electric- and magnetic-field components in the photonic nanostructure with Mie resonators and nanowires; however, this near-unity absorption efficiency could only be achieved over a narrow bandwidth in the range of tens of nanometers as reported in previous works^9,24,25^. In this work, we overcome this narrow bandwidth limitation by designing a new semiconductor metamaterial made of indium gallium arsenide (InGaAs) nanowire metaatoms and precisely arranging them into an array. By optimizing the nanowire array geometry and shape, we design a near-unity absorber over an unprecedented wavelength range, from 400 to 1650 nm, with an average calculated absorptance of 92%. We fabricated the optimized metamaterial design and confirmed its infrared performance by Fourier-transform infrared (FTIR) spectroscopy. The metamaterial exhibited near-unity absorption over the wavelength range from 900 to 1500 nm, with a measured average absorption efficiency of 93% (97% calculated). We compare this measured absorption of the InGaAs nanowire metamaterial to bulk InGaAs of similar thickness and show that the absorptance is significantly enhanced by the nanowire shape and array geometry towards unity. This new class of semiconductor nanowire metamaterials meets the need for a near-unity absorber in the wavelength range where the quantum efficiencies of commercially available detectors are limited without the need for cryogenic cooling. The ability to realize a near-unity absorber in a semiconductor material over such a broad range of wavelengths is of particular interest for developing a next generation single-photon detector for a wider range of applications. Reaching impacts range from quantum technologies in imaging, sensing, communication and computing to biomedical applications such as dose monitoring for cancer treatment and imaging of the eye to identify potentially blinding diseases.

## Mechanisms for enhanced absorption in nanowire metamaterials

**Figure 1:**
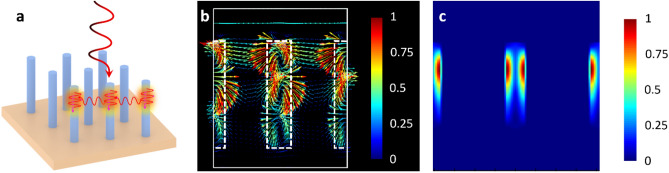
Mechanisms for enhanced absorption. (**a**) Schematic view of the collective interaction of a cylindrical nanowire metamaterial depicting the direct and indirect coupling to leaky guided and transverse modes. The leaky nature of the guided modes in the nanowires leads to free-space scattering of the electric field, which is then recoupled into the neighbouring nanowire metaatoms via excitation of TE and TM modes. (**b**) Simulated electric field response of a nanowire array with periodicity of 833 nm, nanowire diameter of 200 nm and height of 1400 nm. The interaction of the electric field between neighbouring nanowires is depicted by the vectors and their color indicates the field strength. (**c**) Localized absorption profile (indicated by the color) of the nanowire metamaterial at λ = 1020 nm with the same dimensions as (**b**). In the (**b**,**c**) color bars, red represents the maximum magnitude (normalized to 1) and blue is the minimum.

To design a broadband, near-unity absorber, we first investigate the role of periodicity on the absorption profile in cylindrical nanowires. Although high absorption in cylindrical nanowires has been previously investigated^26–28^, the role of periodicity on achieving near-unity absorption has been largely unexplored until now. Figure 1a shows a schematic view of a cylindrical nanowire metamaterial, depicting how leaky resonant modes are being excited in the nanowire array. There are two main avenues for absorption in cylindrical nanowire metamaterials when it is illuminated from the top. First, light can be directly absorbed in the nanowire through coupling to leaky hybrid electric and magnetic modes (HE_11_ and EH_11_)^24,26,27^. Second, light can be indirectly absorbed by scattering from one nanowire to another one through excitation of transverse electric (TE) and transverse magnetic (TM) modes, thus, leading to further absorption. In this latter case, the leaky guided modes migrate towards the nanowire sidewall as they travel downwards. At the interface of the nanowire sidewall and the surrounding dielectric medium, the leaky modes decouple from the nanowire and subsequently emit into free-space. This emitted light then interacts with neighbouring nanowires within the array by coupling to both TE and TM modes. The strong interaction of the electric field between neighboring nanowires for metaatoms with diameter of 200 nm, periodicity of 833 nm and height of 1400 nm is illustrated in Fig. 1b. The resulting spatial absorption profile from these mechanisms in the nanowire metaatoms is shown in Fig. 1c.

The eigenvalue equation, which satisfies Maxwell’s equation for cylindrical boundary conditions, describes the angle dependence of the excited modes in the nanowires^29^. This eigenvalue equation supports the mechanisms of enhanced absorption in nanowire metamaterials as described previously and illustrated in Fig. 1. For all incident angles that are not perpendicular to the nanowire axis, light will couple into HE and EH leaky guided modes. However, when the incident angle of light is perpendicular, purely TE or TM leaky resonant modes are excited. The latter being the transverse modes that are excited in the neighboring nanowires following decoupling of the leaky guided modes.

## Narrowband near-unity absorber—cylindrical nanowire metamaterials

**Figure 2:**
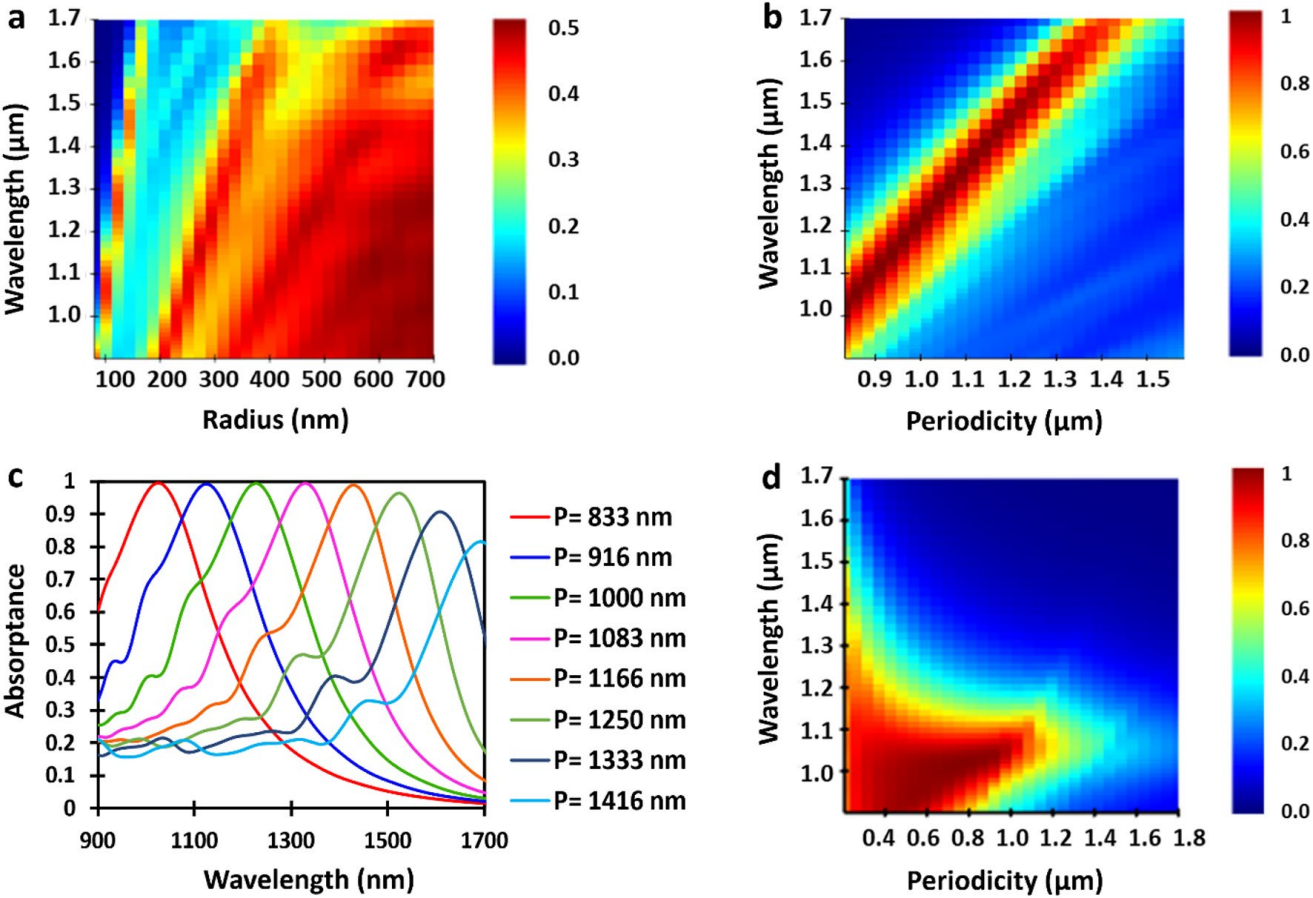
Finite-difference time-domain (FDTD) simulations of the narrowband absorption efficiency for cylindrical InGaAs nanowires. (**a**) Absorption efficiency (color bar) of a single cylindrical nanowire as a function of wavelength and radius. (**b**) Shifting narrowband near-unity absorption efficiency (color bar) response of the nanowire metamaterial as a function of periodicity with a fixed diameter to lattice constant ratio of 0.24. (**c**) Two-dimensional slices of the absorption efficiency from (**b**) showing tunability of the resonant response for near-unity absorption. (**d**) Absorption efficiency (color bar) of a constant 200 nm diameter cylindrical nanowire metamaterial as a function of array periodicity. The nanowire height used in these simulations from (**a**) to (**d**) is 1400 nm.

We further study the cylindrical nanowire case by analyzing the simulated absorptance of a single InGaAs metaatom as a function of its radius (Fig. 2a). For nanowire radii smaller than 175 nm, the absorption profile is narrowband as the nanowire supports a single resonant leaky guided mode only. As the cylindrical nanowire radius increases in this single-mode regime (< 175 nm), the supported resonant leaky modes shift to longer wavelengths. This observation is consistent with what has been previously reported^27^. However, increasing the radius further results in multiple leaky modes to be supported. In this regime of large nanowire radius, multiple leaky modes overlap, and the spectral selectivity vanishes. Thus, the absorption profile broadens as a result. We note that for a larger nanowire radius (> 660 nm), the absorption profile is very similar to the bulk response of the same material. Although the spectral sensitivity in larger nanowire radii vanishes, the peak absorption efficiency of a single nanowire still remains far from unity.

In order to reach near-unity absorption efficiency we place the single nanowire in an array and optimize the periodicity. The role of the array periodicity on the absorption efficiency of a nanowire metamaterial has not yet been fully explored until now. Our analysis shows that the absorption efficiency of the cylindrical semiconductor nanowire metamaterial is directly linked to the array periodicity. The results of our analysis are summarized in Fig. 2b,c. Figure 2b shows the calculated absorptance for varying nanowire periodicity and incident wavelength, while Fig. 2c takes two-dimensional slices of this three-dimensional plot from Fig. 2b. Here, we find the maximum absorptance with the narrowest bandwidth occurs at the nanowire diameter to periodicity ratio of 0.24. Remarkably, by maintaining this ratio for varying lattice constant (periodicity), near-unity absorption efficiency (> 99%) can be selectively achieved in the wavelength range from approximately 1000 to 1400 nm (see Fig. 2c). Furthermore, minimal roll-off in the absorption efficiency as the incident wavelength approaches the InGaAs bandgap is observed (~ 96% at 1500 nm and ~ 80% at 1650 nm).

To better understand the underlying mechanisms contributing to near-unity absorption in the nanowire metamaterial, we focus our attention on nanowires with dimensions: 200 nm diameter and 1400 nm height. A nanowire height of 1400 nm was selected since this was close to the smallest height where broadband near-unity absorptance was achieved (see Supplementary Note 4). The calculated absorption efficiency with these nanowire dimensions as a function of periodicity is presented in Fig. 2d. Near-unity absorption (> 99%) occurs for a range of periodicities between 676 and 885 nm. In this range of nanowire periodicities, the process of indirect absorption attributed to the decoupling of leaky resonant guided modes and excitation of TE or TM modes in neighbouring nanowires is enhanced. This enhancement maximizes the overall absorption efficiency and highlights its dependence on the nanowire periodicity. Interestingly, this process of near-unity absorption only occurs when the nanowire spacing is optimized. When deviating from this optimized spacing the spectral sensitivity vanishes and the absorption efficiency drops. A smaller nanowire periodicity leads to a broadened absorption spectrum with a lower maximum. In contrast, the absorption spectrum remains narrow for a larger nanowire periodicity, but the maximum gradually drops as the interaction between neighbouring nanowires diminishes.

## Broadband near-unity absorber—tapered nanowire metamaterial

While cylindrical nanowire metamaterials offer narrow bandwidth, near-unity absorption capabilities, certain sensing applications require broad spectral range detection. One successful approach with a nanowire metamaterial utilizes tapered nanowire metaatoms to accommodate the coupling of a broad range of frequencies^26,30–33^. In this strategy, the nanowire tapering provides a continuum of diameters for all wavelengths to couple to resonant HE and EH leaky guided modes. This broadband behaviour combined with a proper choice of semiconductor material can produce a desirable broadband metamaterial absorber in the infrared.

**Figure 3:**
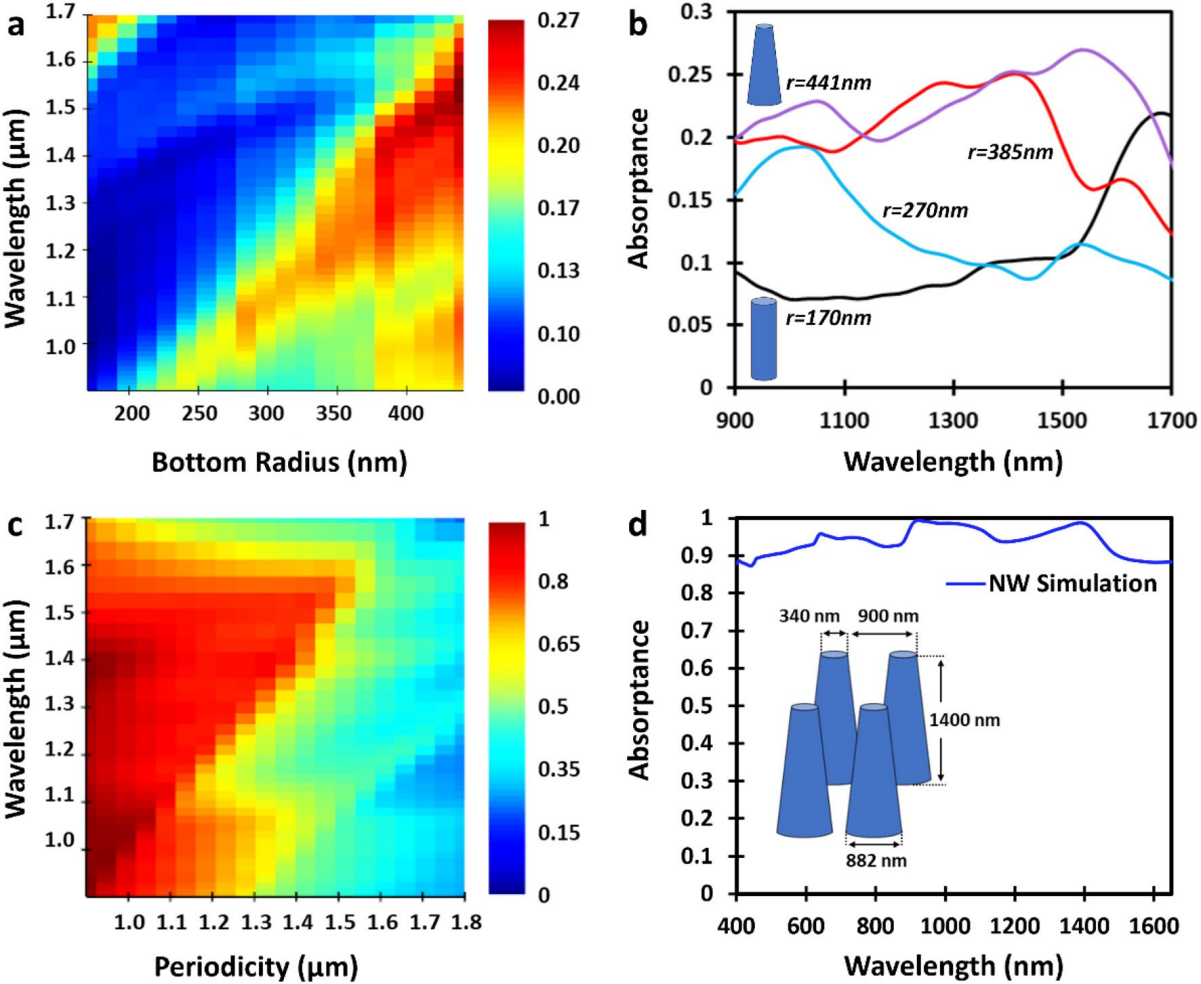
Finite-difference time-domain (FDTD) simulations of the broadband absorption efficiency for tapered InGaAs nanowires. (**a**) Calculated absorption efficiency (color bar) of a single tapered nanowire as a function of bottom nanowire radius with a fixed top radius of 170 nm and height of 1400 nm. (**b**) Two-dimensional slices of the absorption efficiency from (**a**) of a single nanowire with varying bottom radius (170 nm, 270 nm, 385 nm, 441 nm) and a fixed top radius of 170 nm. The nanowire is cylindrical at r = 170 nm as indicated by the bottom inset. Increasing the bottom radius leads to a nanowire tapering as indicated by the top inset for r = 441 nm. (**c**) Calculated absorption efficiency (color bar) dependence on the nanowire periodicity for a nanowire height of 1400 nm, top radius of 170 nm and bottom radius of 441 nm. (**d**) Calculated absorption efficiency over an extended wavelength range for an optimized nanowire metamaterial, demonstrating near-unity absorption over an unprecedented wavelength range from 400 to 1650 nm. The optimized dimensions (shown in the inset) were found to be: lattice constant: 900 nm; bottom diameter: 882 nm; top diameter: 340 nm; and height: 1400 nm.

To design a near-unity broadband absorber in the infrared, we first optimize the tapered InGaAs nanowire shape of a single nanowire by varying the bottom radius for a fixed top radius. The top radius was selected (r = 170 nm) to coincide with the cylindrical nanowire radius that produced the highest resonant spectral response near the edge of the InGaAs bandgap (1650 nm). The bottom radius of the tapered nanowire was increased in size until the absorption was enhanced over a broad spectral range (see Fig. 3a,b). The optimal combination of parameters that we found included a top nanowire radius of 170 nm, bottom nanowire radius of 440 nm and height of 1400 nm.

Next, the optimal nanowire metaatom from Fig. 3a was placed in a two-dimensional square lattice to form a metamaterial where the lattice constant can be leveraged to achieve broadband, near-unity absorption. In Fig. 3c, we plot the calculated absorption efficiency as a function of periodicity. For this metaatom, we determine that broadband, near-unity absorption in the metamaterial is achieved at a lattice spacing of 900 nm. Increasing the periodicity leads to a decrease in the absorption efficiency as the interaction between neighbouring nanowires weakens. Similar to cylindrical nanowire metamaterials, this behaviour implies that the larger lattice constant reduces the effective recollection of the scattered field by the tapered nanowire array. We note that a lower bound of 900 nm was set for the periodicity since a smaller separation would result in the nanowires overlapping, reducing their selected height. A more detailed description of the nanowire metamaterial optimization process is presented in Supplementary Notes 1–5.

**Figure 4:**
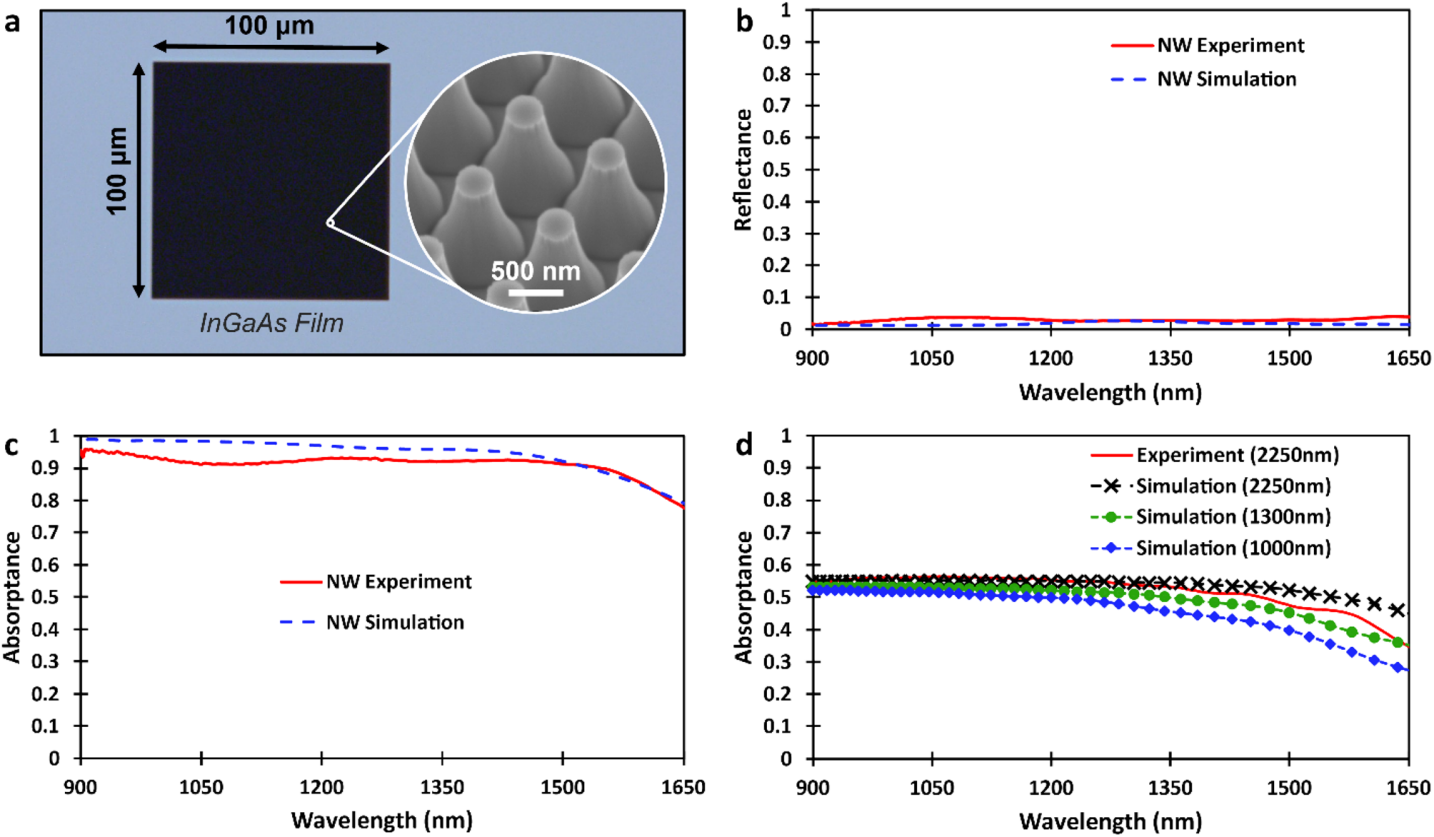
Near-unity nanowire metamaterial absorption. (**a**) Optical microscope image of the fabricated InGaAs tapered nanowire metamaterial (black square) on an InGaAs film (blue-grey). The lack of contrast in the 100 μm × 100 μm active area is indicative of high absorption. The inset shows a scanning electron micrograph of tapered InGaAs nanowires that is representative of the selected area from the high absorbing metamaterial region. (**b**) Comparison of the simulated and experimentally measured reflectance for the nanowire metamaterial as a function of wavelength. The fabricated (modelled) nanowire metamaterial dimensions are similar with a top radius: 175 nm (170 nm), bottom radius: 440 nm (441 nm), height: 1300 nm (1400 nm), and a pitch: 900 nm (900 nm). (**c**) Comparison of the simulated and experimentally measured absorption spectra of an InGaAs tapered nanowire metamaterial with the same dimensions as (**b**), which demonstrates the near-unity absorption over an unprecedented wavelength range. The measured (simulated) average absorption efficiency from 900 to 1500 nm is 93% (97%). (**d**) Measured and calculated absorption spectra of a planar InGaAs film on an InP substrate for various thicknesses—measured film: 2250 nm; modelled films: 1000 nm, 1300 nm, and 2250 nm.

In Fig. 3d, we plot the calculated absorption efficiency over an extended wavelength range (400–1650 nm) at the optimal periodicity to highlight near-unity absorption over an unprecedented bandwidth. The calculated average absorption efficiency is found to be 92%, with peak efficiencies of 99% at 909 nm and 98% at 1406 nm. We note that the peak at 909 nm corresponds to the 900 nm periodicity of the nanowire array (resonant lattice mode). The other peaks observed in Fig. 3d are attributed to operating in the multimode regime and the nanowires being suspended in air. These peaks are found to be less prominent in our updated model which includes a substrate to better compare with the experimental results in Fig. 4.

## Results

Next, we fabricate the nanowire metamaterial absorber with dimensions as close as possible to the optimized structure using an InGaAs film (2250 nm thickness) grown by molecular beam epitaxy on an (100) InP substrate. We utilized a multistep nanofabrication process to realize a 100 μm × 100 μm metamaterial comprised of a tapered InGaAs nanowire array. See Supplementary Note 6 for details on the nanowire fabrication process.

A scanning electron micrograph of the fabricated optimized nanowire metatoms is shown in the inset of Fig. 4a. This image illustrates the uniformity of the tapered nanowires that was achieved during the fabrication process. The high absorption efficiency of the nanowire metamaterial is apparent in the optical image of Fig. 4a as the array appears black when compared to the blue-grey InGaAs film. We performed FTIR spectroscopy to measure the absorption efficiency of the nanowire array. A tungsten light source was used to illuminate the sample, and a liquid-nitrogen-cooled Mercury-Cadmium-Telluride (MCT) detector was employed to measure the intensity of the reflected and transmitted spectra. To achieve a spot size smaller than the nanowire metamaterial dimensions in these measurements, a Schwarzschild reflective objective with a numerical aperture of 0.5 was used. The measured reflectance as a function of wavelength is shown in Fig. 4b, illustrating the low reflection from the nanowire metamaterial. The reflectance is found to be below 4% over the entire wavelength range from 900 to 1650 nm, with an average reflectance of 3%. These results are compared to simulations of the same nanowire metamaterial dimensions, which show excellent quantitative agreement. We note that the fabricated nanowire metamaterial dimensions closely resemble the optimized structure from the numerical calculations. The fabricated nanowire metamaterial (modelled metamaterial) was found to approximately have a top radius of 175 nm (170 nm), bottom radius of 440 nm (441 nm), height of 1300 nm (1400 nm), and a pitch of 900 nm (900 nm). We therefore updated the dimensions in our model for a direct comparison with experiment. We also note that the reflective objective in the FTIR experimental setup introduces a range of angles for light incident on the nanowire array from normal (0°) to 30°. Additionally, the nanowire array is fabricated from an InGaAs film on an InP substrate; however, up to this point the numerical models only considered InGaAs nanowires suspended in air. Thus, these factors were accounted for in our improved model for a more realistic comparison (further details in Supplementary Notes 3 and 4).

We now bring attention to the measured broadband near-unity absorption, as shown in Fig. 4c. Remarkably, we measure an average absorptance of 93% from 900 to 1500 nm, with a peak efficiency of 95%. Comparing these experimental results with the simulated data in Fig. 4c, an excellent agreement is attained with minimal discrepancy. The small deviation that is observed between simulation and experiment may be due to the differing sidewall geometries of the fabricated and modelled nanowires. To obtain the absorption efficiency from the measured results, we utilize A = 1 − R − T, where A, R and T are the absorptance, reflectance, and transmittance, respectively. In our measurement, we found that the transmission data also comprises of thin film interference due to the presence of the reflective objective. We therefore corrected for this interference in the measured absorptance. Details of this correction procedure is presented in Supplementary Note 8. We note that the process we implemented to correct for the thin-film interference underestimates the measured absorptance, thus providing a conservative lower bound to the absorption efficiency of the nanowire metamaterial.

To demonstrate the enhanced absorption in tapered nanowire metamaterials we compared it to an unprocessed bulk InGaAs film with a thickness of 2250 nm. The measured bulk InGaAs film absorptance is shown as a function of wavelength in Fig. 4d (red solid curve). This experimental data is compared to the simulated bulk InGaAs film with an identical thickness of 2250 nm and demonstrates excellent agreement (black dashed cross). The enhancement of the absorption towards unity when utilizing the tapered nanowire geometry is evident when comparing tapered nanowires (Fig. 4c) and the planar bulk material (Fig. 4d). We also simulated bulk InGaAs with an identical thickness (1300 nm) to the thickness of the tapered nanowire array (green dashed circle) and a thickness (1000 nm) of commercially available InGaAs photodetectors for reference (blue dashed diamond). Numerical calculations and the measured performance for a series of varied nanowire geometries were also studied (see Supplementary Note 10). Despite deviating from the optimized structure, our measurements show the reproducibility of enhanced absorption in tapered nanowire metamaterials.

## Conclusion

We studied the theoretical mechanisms that govern the enhanced absorption in nanowire metamaterials, with emphasis in the infrared. We identified two prominent mechanisms that contribute to near-unity absorption in nanowire metamaterials. These include the free-space coupling of photons into the nanowires which excite leaky guided modes, followed by the strong interaction between neighbouring nanowires which excite transverse modes. We subsequently showed the importance of nanowire periodicity on achieving the maximum absorption efficiency of the metamaterial by manipulating the interaction between neighbouring nanowires. We optimized for near-unity absorption in cylindrical and tapered nanowire metamaterials, demonstrating a systematic approach to achieve narrowband and broadband characteristics. Furthermore, we fabricated a tapered InGaAs nanowire metamaterial with dimensions similar to the optimized structure. With this optimized structure, we experimentally demonstrated the near-unity absorption efficiency over an unprecedented wavelength range from 900 to 1500 nm, with an average efficiency of 93%. Although we demonstrated near-unity broadband absorption of the nanowire metamaterial in the near-infrared region, our approach is not limited to only InGaAs. The nanowire metamaterial can be tailored to achieve enhanced absorption in the mid-infrared and other spectral regions through the incorporation of appropriate semiconductor materials. Realizing this approach to achieve near-unity absorption efficiency over such a broad bandwidth for the first time will play a major role in the discovery of a new generation of photodetectors targeting a wider range of applications from biomedical to quantum information.

## Supplementary Information

Supplementary Information.

## Data Availability

The data that support the plots within this paper and other findings of this study are available from the corresponding author upon reasonable request.
