# Supplementary material for: Semiconductor nanowire metamaterial for broadband near-unity absorption
Source: Sci Rep. 2022 Jun 11;12:9663. doi: 10.1038/s41598-022-13537-y (PMC9188558; doi:10.1038/s41598-022-13537-y)
Supplement: Supplementary file 1 — Supplementary Information. [file 41598_2022_13537_MOESM1_ESM.pdf]

Semiconductor nanowire metamaterial for broadband near-unity absorption

[†]Burak Tekcan[1,2], [†]Brad van Kasteren[1,2], [†]Sasan V. Grayli[1,2], Daozhi Shen[1,4,5], Man Chun Tam[2,3], Dayan Ban[2,3], Zbigniew Wasilewski[2,3], Adam W. Tsen[1,3,4,6], Michael E. Reimer[1,2,6*]

[1]Institute for Quantum Computing, University of Waterloo, Ontario, Canada. [2]Department of Electrical & Computer Engineering, University of Waterloo, Ontario, Canada. [3]Waterloo Institute for Nanotechnology, University of Waterloo, Ontario, Canada. [4]Department of Chemistry, University of Waterloo, Ontario, Canada. [5]Centre for Advanced Materials Joining, University of Waterloo, Ontario, Canada, [6]Department of Physics and Astronomy, University of Waterloo, Ontario, Canada.
†These authors contributed equally to this work.

SUPPLEMENTARY 1: General Numerical Calculation Implementations

The modeling of the nanowire metamaterial devices that are presented in the manuscript are all done using the Finite-Difference Time-Domain (FDTD) Lumerical Solutions. The optical absorption characteristics of the structures were calculated using the advanced absorption analysis group provided by the modeling tool. The models were created with a fine mesh size relative to the structures (0.025 nm). For non-repeating structures, a Total-Field Scattered-Field (TFSF) source was used throughout the optimization process with perfect matched layer (PML) boundary conditions. The numerical calculations for the nanowire metamaterial arrays were run with a planewave source and Periodic Boundary Conditions (PBC).

SUPPLEMENTARY 2: Tapered Nanowire Dimension Optimization Process

The dimensions of the tapered nanowires were optimized by first finding a cylindrical nanowire diameter that supports a single leaky resonant mode at the energy close to the bandgap of InGaAs at room temperature ($E_g \approx 0.75$ eV, $\lambda \approx 1650$ nm). FDTD Lumerical solutions was used to model the absorption characteristics of the tapered nanowire by systematically increasing the bottom diameter of the cylinder until the highest performance broadband absorption over the target spectral region was

achieved. Then, the optimized tapered nanowire was placed in a periodic lattice structure and the spacing between the nanowires (periodicity) was swept over the target range of 900 nm to 1800 nm. The absorption efficiency as a function of wavelength vs. periodicity was plotted and the lattice spacing with highest broadband absorption for the target spectral range was selected.

The initial optimization was done with air as the background medium (n=1) and without a substrate. In this way, the nanowire array was suspended in air and remained the only contributor to the targeted absorption being maximized. The height of the nanowires was set to 1400 nm based on insights from previous numerical calculations of the nanowire metamaterial.

SUPPLEMENTARY 3: Improved Model Accuracy of the Metamaterial with Tapered Nanowire Metaatoms

In order to verify the agreement between the experimentally measured absorption spectra and the calculated results from simulation, the optical absorption characteristics were obtained by measuring the absorption spectra of InGaAs nanowires fabricated on InGaAs/InP substrate. The incoming light passes through a Schwarzschild reflective objective with a numerical aperture of 0.5, introducing incident angles from -30° to 30° of the focused beam. This was accounted for in the numerical calculations by sweeping the source angle of incidence over a matching range and averaging the absorptance.

SUPPLEMENTARY 4: Effect of Tapered Nanowire Height on the Metamaterial Absorption Efficiency

We investigated the effect of the metaatom nanowire height on the absorption efficiency by systematically reducing the nanowire height from 1.6 µm to 200 nm (Figure S1a). The lattice structure was constructed from the tapered nanowires infinitely repeating with periodicity of 900 nm without a

substrate. The top and bottom diameters match the fabricated metamaterial absorbers, i.e., the top diameter is 350 nm and bottom diameter is 880 nm. It was observed that the high-performance broadband absorption efficiency over the target spectrum (0.9 µm to 1.7 µm) remains relatively unchanged for nanowire heights of 1.2 µm to 1.6 µm. As the height of the nanowires are reduced below 1.2 µm, the absorption efficiency performance is shown to noticeably decrease.

Next, the effect of the nanowire height was studied after including a substrate in the numerical calculation to reflect a more realistic comparison between simulations and the fabricated nanowire arrays. The substrate consisted of an InP wafer and a thin film of InGaAs to match the parameters of the fabricated metamaterials. The absorption response of the updated metamaterial was then found as a function of nanowire height over the high broadband performance height range found for the suspended nanowires (1 µm to 1.6 µm). The thickness of the total InGaAs (including the nanowire and substrate thickness) film remained constant, i.e., the substrate thickness was increased to maintain a total thickness of 2250 nm of the InGaAs film when the height of the nanowire was decreased. As seen in Figure S1b, the absorption efficiency showed little dependence on the changing nanowire height when the substrate is included, highlighting the impact of the substrate on the overall metamaterial absorption efficiency characteristics.

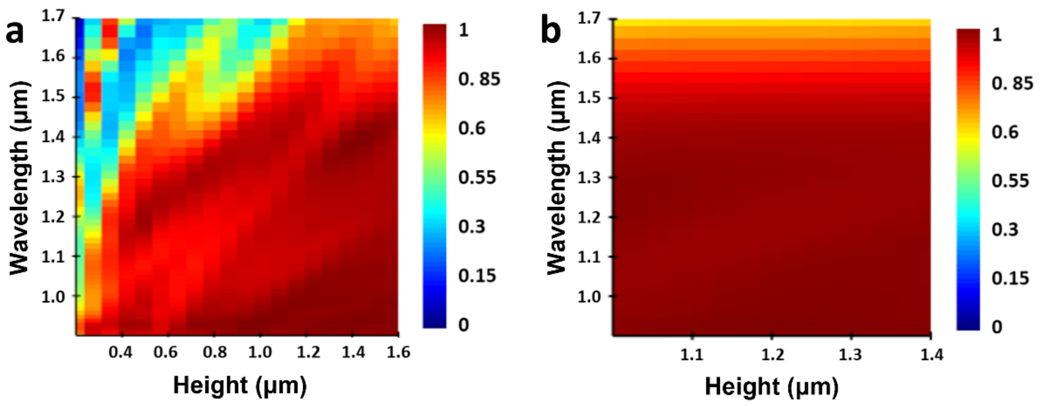

Figure S1. The effect of height on the absorption response of the InGaAs tapered nanowire metamaterial. The height absorption response is shown without a substrate in a) and with a substrate in b). Without the substrate, the impact of the changing metaatom height becomes apparent in the absorption efficiency of the metamaterial.

Once the nanowire height is reduced below 1 µm, the high broadband absorption performance of the metamaterial begins to diminish. b) With the substrate, the broadband absorption efficiency of the metamaterial does not undergo a noticeable change with the varying nanowire height and when compared to a).

SUPPLEMENTARY 5: Growth and Characterization of the Indium Gallium Arsenide Film

The InGaAs film that was used to fabricate the metamaterial array was grown by a Veecco GEN10 molecular beam epitaxy system, located at the Quantum Nano Centre Molecular Beam Epitaxy Lab (University of Waterloo), on a (100) InP single crystal wafer. Ellipsometry was used to extract the complex refractive index of the InGaAs film and is plotted in Figure S2. This optical data was imported into the Lumerical FDTD software and it was further used in all of the InGaAs nanowire metamaterial modeling.

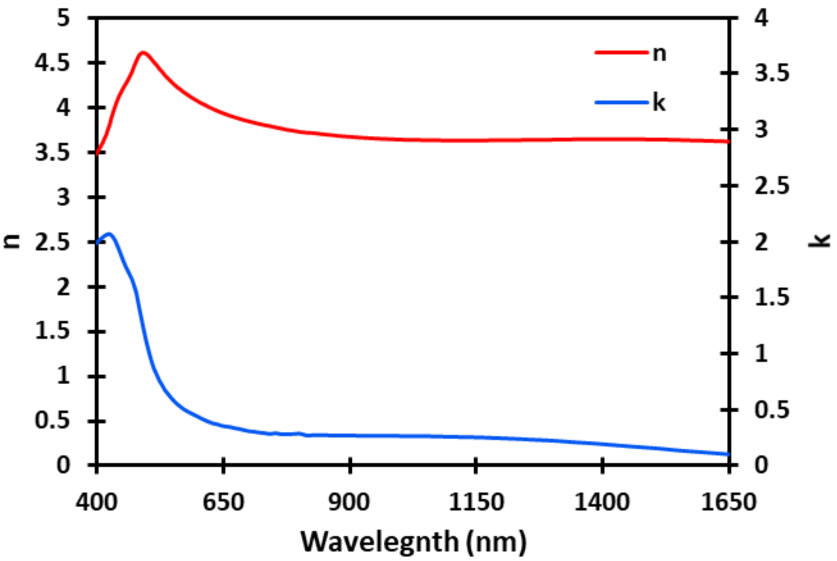

Figure S2. Complex refractive index of InGaAs. The spectroscopic ellipsometry measured complex refractive index of the epitaxially grown InGaAs film on a (100) InP single crystal wafer.

SUPPLEMENTARY 6: Fabrication of the InGaAs Tapered Nanowire Metamaterial

The tapered nanowire arrays fabrication process started with the growth of a planar InGaAs epitaxial layer on a commercial InP substrate by Molecular Beam Epitaxy (MBE). The planar InGaAs thin films were cleaved into 0.75 x 0.75 cm square chips and cleaned by Acetone and IPA 5 minutes each in succession in

ultrasonic tanks. The cleaned chips were loaded in a Plasma Enhanced Chemical Vapor Deposition (PECVD) system for SiNx deposition. A 500 nm thick SiNx film was deposited as a hard mask for the nanowire etch stage. Next, the chips were spun with PMMA A3 (~150nm), followed by electron beam lithography to initialize the nanowire array patterning. The chips were developed with MIBK:IPA (1:3) for 60 seconds before depositing 15 nm Al:40 nm Cr. The Al:Cr metal layer was used to transfer the e-beam lithography pattern onto the SiNx hard mask. Then, the SiNx hard mask array pattern was created by etching SiNx in an Inductively Coupled Plasma Reactive Ion Etching (ICP-RIE) system to achieve a high aspect ratio hard mask pattern. Next, the InGaAs layers were etched down to a desired depth using the ICP-RIE system. Finally, the InGaAs etched samples were dipped into a 10:1 Buffered Oxide Etchant (BOE) solution for 7 minutes to remove the remaining SiNx hard mask caps. Figure S4a illustrates the fabrication process flow of the tapered InGaAs nanowire array broadband absorber. Figure S4b and S4c show scanning electron micrographs of the fabricated InGaAs nanowire arrays.

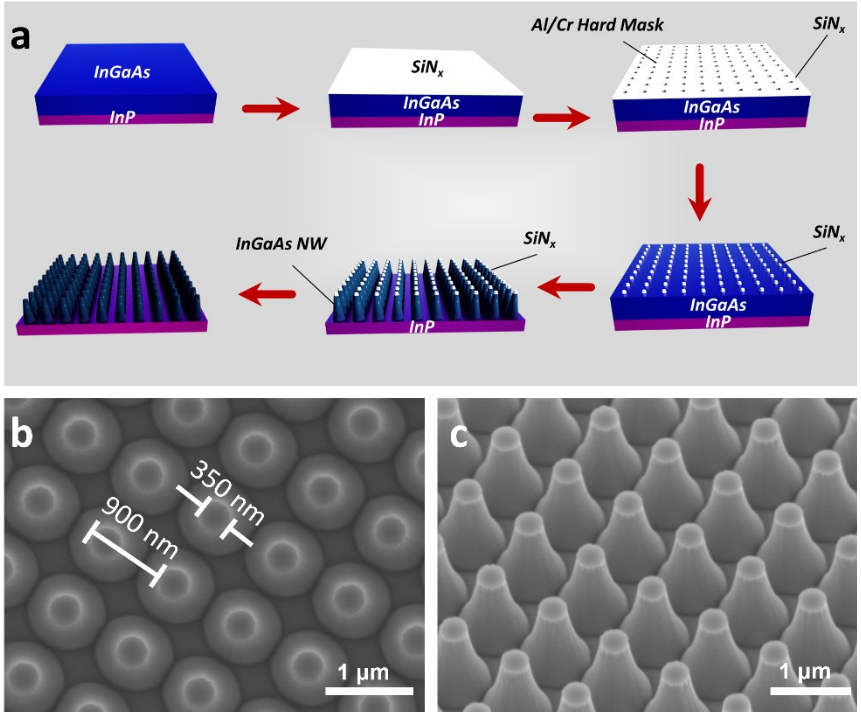

Figure S3. Fabrication of the nanowire metamaterial. The fabrication steps of the InGaAs tapered nanowire array are shown in a). b) and c) are the scanning electron micrograph of the fabricated nanowires with estimated periodicity of 900 nm and the top diameter of 350 nm.

SUPPLEMENTARY 7: FTIR Measurement

The transmission and reflection spectra of the samples were measured using a Bruker IFS 66v/S Fourier-transform infrared (FTIR) spectrometer. A tungsten halogen source (OSRAM, 275W) was focused onto the sample under test by a 36× reflective objective (Ealing, NA = 0.5), following the FTIR beam splitter. For the transmission spectrum measurement, the transmitted light from sample and background was collected by another reflective objective (behind the sample) and directed to a liquid-nitrogen-cooled mercury cadmium telluride (MCT) detector (EG&G JUDSON J16D-M204-R05M-60). For the reflection measurement, a D-shaped mirror (Thorlabs PFD10-03-P01) was placed before the Schwarzschild reflective objective. The reflected light from sample was collected into the MCT detector by the D shape mirror. The D-shaped mirror used in this reflection measurement was a silver mirror (Thorlabs PF10-03-P01) with a reflectivity > 97%, this was used as the reference spectrum for the wavelength range of interest.

SUPPLEMENTARY 8: FTIR Data Processing

The measured reflection/transmission spectra were used to extract the absorption characteristics of the fabricated metamaterial samples. Reference data was collected for the source, absent of any absorber samples. This reference data was used to normalize the FTIR data collected from the sample measurements to the source. The sample absorption efficiency was then calculated from the normalized FTIR sample data, knowing $A = 1 - T - R$. Where A is the absorptance, T is the transmittance, and R is the reflectance. The resulting absorption data was then adjusted to account for the artificial absorption observed below the InGaAs bandgap energy, which resulted from the thin film interference in the transmission data that was introduced from the reflective objective. To account for this interference, the data was scaled to be considerate of the InGaAs bandgap where the lowest absorption is expected

to be near zero. The background correction method used scaled the absorption data according to the formula $(x_i - x_{min})/(1-x_{min})$. The results from scaling each data point by this formula bounded the lower absorptance to near zero and underestimates the absorption efficiency of the nanowire metamaterial. Despite the conservative approach, the resulting metamaterial absorption data presented an average of 93% absorption efficiency between 900 nm-1500 nm range. The absorption measurement was plotted before and after the correction in Figure S2. Here, the arrow indicates the region below bandgap where the scaled absorptance was lower bounded to near zero.

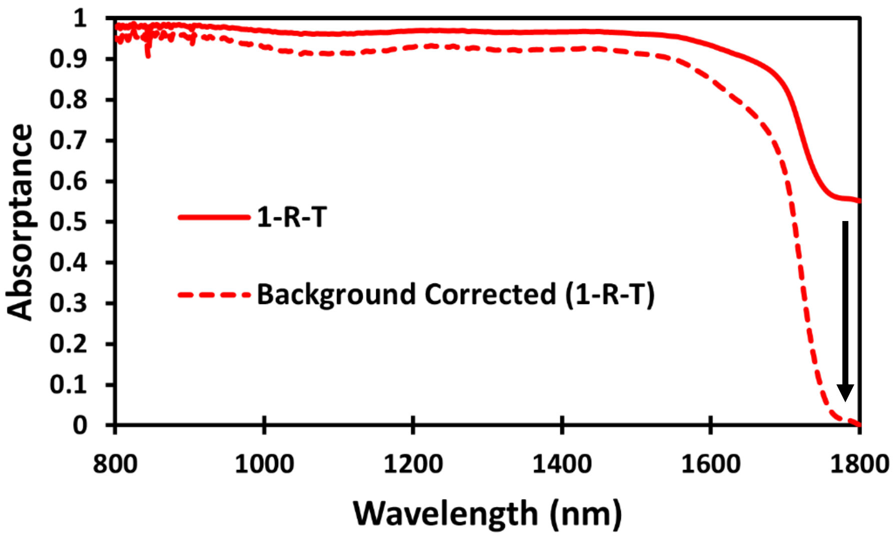

Figure S4. The calculated absorption efficiency of the fabricated nanowire metamaterial before and after the thin film interference background correction. This plot was obtained from subtracting the source normalized sample reflection and transmission data from unity. The arrow indicates the region below the InGaAs bandgap which was expected to exhibit a near zero absorption.

SUPPLEMENTARY 9: Planar InGaAs FTIR Measurement Process

The optical absorption characteristics of a planar unprocessed InGaAs film (thickness=2250 nm) was obtained according to the FTIR measurement process described in the "FTIR Measurement" section. The experimental setup for the planar measurements differed slightly, however. The reflection and transmission data for the planar samples were collected without the use of a reflective objective; the planar sample was illuminated with a semi-collimated beam instead to prevent the uncertainty that

needed to be adjusted for in the data observed in the nanowire sample measurements (see section “FTIR Data Processing”). The planar absorption measurement was compared with the modeled absorption response of 2250 nm, 1400 nm and 1000 nm thick InGaAs films of manuscript Figure 4d.

SUPPLEMENTARY 10: Optical Properties of the Tapered Nanowire Metamaterials with Varying Metaatom Unit Cells

To study the success of the optimization process and simulation model, additional nanowire metamaterial designs were numerically and experimentally analyzed. This process also acted to further improve understanding for how the physical properties of the metaatoms can impact the metamaterial broadband absorption behaviour. Specifically, a series of tapered nanowire metamaterials with varying pitch and radii were investigated. The scanning electron micrographs of the various nanowire metamaterials, corresponding to NW1-NW4, are included in Figure S5. The broadband absorption responses of these various metamaterials were also experimentally assessed with FTIR, according to the “FTIR Measurement” and “FTIR Data Processing” sections. Figure S6 shows the corresponding modeled and experimentally obtained broadband absorption response of the fabricated nanowire metamaterials NW1 – NW4. We note that the metaatom nanowire height for NW1-NW4 was approximately 1.3 μm.

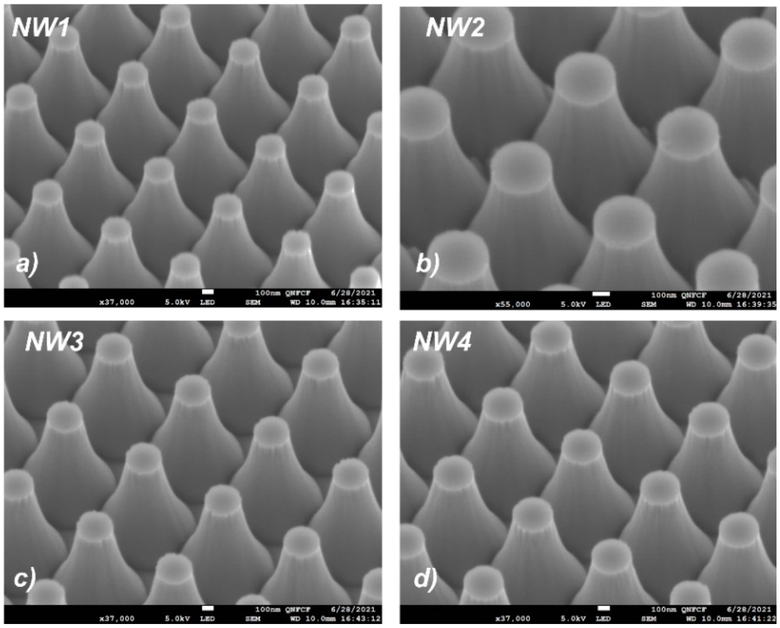

Figure S5. The tilted angle scanning electron micrographs of the fabricated metamaterials NW1-NW4. a) Sample NW1 was measured to have: d_top = 250 nm, d_bottom = 682 nm, periodicity = 700 nm. b) Sample NW2: d_top = 350 nm, d_bottom = 682 nm, periodicity = 700nm. c) Sample NW3: d_top = 300nm, d_bottom = 782nm, periodicity = 800nm. d) Sample NW4: d_top = 350 nm, d_bottom_782 nm, periodicity = 800 nm. The height of the nanowires in all of the fabricated metamaterials was measured to be approximately 1.3 µm.

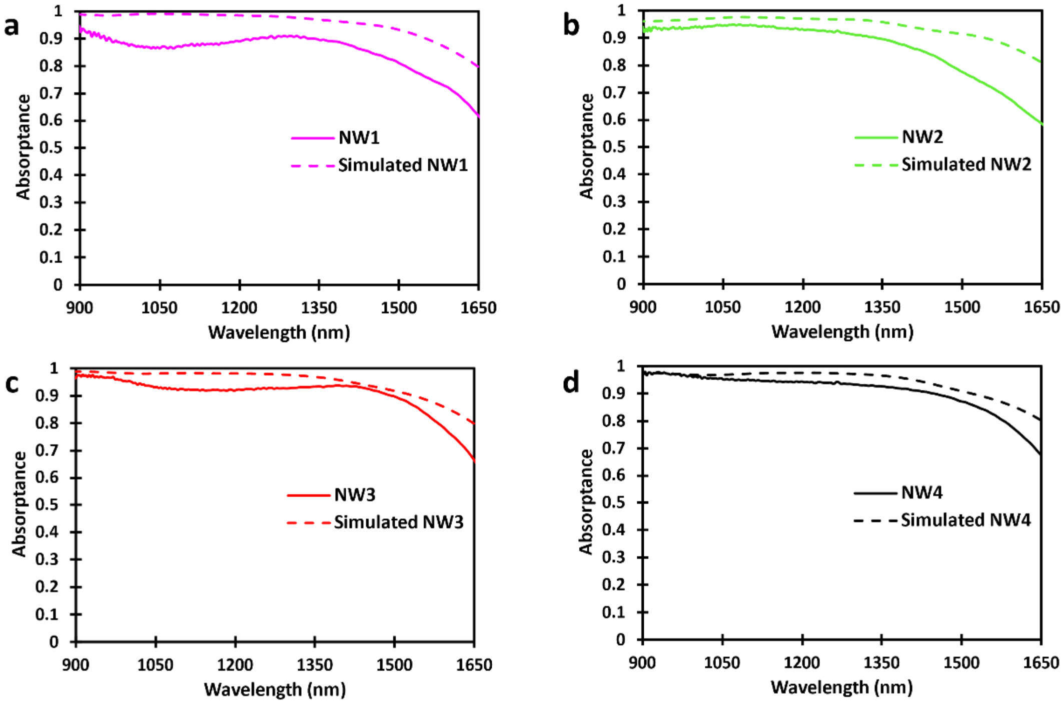

Figure S6. The numerical and FTIR experimental absorption efficiency of four different broadband nanowire metamaterial absorbers, NW1-NW4. a)-d) show the measured and modeled absorption efficiencies of the respective metamaterials outlined in Figure S5.
